# Sequence analysis and protein interactions of Arabidopsis CIA2 and CIL proteins

**DOI:** 10.1186/s40529-020-00297-z

**Published:** 2020-06-18

**Authors:** Chun-Yen Yang, Chih-Wen Sun

**Affiliations:** Department of Life Science, National Taiwan Normal University, Taipei, 116 Taiwan

**Keywords:** CIA2, CIL, Nuclear localization signal, Yeast two-hybrid

## Abstract

**Background:**

A previous screening of *Arabidopsis thaliana* for mutants exhibiting dysfunctional chloroplast protein transport identified the *chloroplast import apparatus* (*cia*) gene. The *cia2* mutant has a pale green phenotype and reduced rate of protein import into chloroplasts, but leaf shape and size are similar to wild-type plants of the same developmental stage. Microarray analysis showed that nuclear CIA2 protein enhances expression of the *Toc75*, *Toc33*, *CPN10* and *cpRPs* genes, thereby up-regulating protein import and synthesis efficiency in chloroplasts. CIA2-like (CIL) shares 65% sequence identity to CIA2, suggesting that CIL and CIA2 are homologous proteins in Arabidopsis. Here, we further assess the protein interactions and sequence features of CIA2 and CIL.

**Results:**

Subcellular localizations of truncated CIA2 protein fragments in our onion transient assay demonstrate that CIA2 contains two nuclear localization signals (NLS) located at amino acids (aa) 62-65 and 291-308, whereas CIL has only one NLS at aa 47-50. We screened a yeast two-hybrid (Y2H) Arabidopsis cDNA library to search for putative CIA2-interacting proteins and identified 12 nuclear proteins, including itself, CIL, and flowering-control proteins (such as CO, NF-YB1, NF-YC1, NF-YC9 and ABI3). Additional Y2H experiments demonstrate that CIA2 and CIL mainly interact with flowering-control proteins via their N-termini, but preferentially form homo- or hetero-dimers through their C-termini. Moreover, sequence alignment showed that the N-terminal sequences of CIA2, CIL and NF-YA are highly conserved. Therefore, NF-YA in the NF-Y complex could be substituted by CIA2 or CIL.

**Conclusions:**

We show that Arabidopsis CIA2 and CIL can interact with CO and NF-Y complex, so not only may they contribute to regulate chloroplast function but also to modulate flower development.

## Background

Plant chloroplasts are differentiated from proplastids and are responsible for photosynthesis, synthesis of amino acids, lipids, and phytohormones, and storage of starch and oil compounds during plant growth and development. According to the endosymbiont hypothesis, chloroplasts became incorporated into plant cells by endocytosis of cyanobacteria. Most cyanobacterial genes were then transferred into the host nucleus over the course of evolution (Martin et al. 2002; Imamura et al. 2009; Pfalz and Pfannschmidt, 2013). Chloroplasts have 3100 proteins, more than 90% of which are encoded by nuclear genes and are transferred into chloroplasts (Leister, 2003; Pfalz and Pfannschmidt, 2013; Paila et al. 2015), apart from a small number translated from the chloroplast genome. Therefore, proteins involved in chloroplast development and other vital functions require cooperation of genes in both the nucleus and chloroplast.

*Arabidopsis thaliana* mutant screening (using ethyl-methane sulfonate mutagenesis and antibiotic resistance screening methods) for lines defective in chloroplast protein transport identified the *chloroplast import apparatus* (*cia*) gene. The *cia2* mutant line has a pale green phenotype and exhibits a ~ 50% reduction in total chlorophyll and carotenoid contents relative to wild-type plants. However, leaf shape and size are similar to wild-type plants of the same developmental stage (Sun et al. 2001). Moreover, chloroplast protein import efficiency is lower in *cia2* mutant relative to wild-type (Sun et al. 2001). Microarray analysis showed that CIA2 enhances the expression of the *translocon at the outer envelope membrane of the chloroplast 75* (*Toc75*)*, Toc33, chaperonin10* (*CPN10*) and chloroplast *ribosomal protein* (*cpRP*) genes. Thus, CIA2 up-regulates protein import and synthesis efficiency in chloroplasts (Sun et al. 2009).

*CIA2* encodes a nuclear protein of 435 aa. The *cia2* mutant exhibits a G-to-A mutation at nucleotide 770 that converts a tryptophan residue into a stop codon at aa 257, resulting in a truncated CIA2 protein (Sun et al. 2001). Arabidopsis *CIA2*-*like* (*CIL*) encodes a 394-aa protein with 65% sequence identity to CIA2. Expression of the *CIL* transcript is enhanced in the *cia2* mutant, indicating that CIL might be functionally redundant to CIA2 (Sun et al. 2001). Both CIA2 and CIL have a 43-aa CCT [CONSTANS (CO), CO-LIKE (COL), and TIMING OF CAB EXPRESSION 1 (TOC1)] (Putterill et al. 1995; Gangappa and Botto, 2014) motif at their C termini (Sun et al. 2001). CCT motifs are reported to function as nuclear localization signals (NLS) and as a protein–protein interaction region for CO, COL and TOC1 (Kurup et al. 2000; Strayer et al. 2000; Robson et al. 2001). However, whether the CCT motifs of CIA2 and CIL retain those functions was unknown.

CCT motif-containing proteins are classified into three subfamilies according to their N-terminal structures, i.e., COL, pseudo-response regulator (PRR) and CCT-motif family (CMF). The N termini of COL and PRR proteins have a B-box domain and a PRR domain, respectively. However, the N terminus of CMF proteins does not contain any specific structure or a known domain (Cockram et al. 2012). COL proteins are mainly involved in regulating photoperiodic flowering (Putterill et al. 1995; Yano et al. 2000; Turner et al. 2005). PRR proteins modulate circadian rhythms and light-signaling transduction (Salomé et al. 2006; Nakamichi et al. 2010, 2012). The functions of CMF proteins in plant cells have yet to be fully ascertained. Two Arabidopsis CMF proteins, named ASML2 (or CMF8) and CIA2 (or CMF14), have been shown to differentially regulate the expression of sugar-inducible genes (Masaki et al. 2005) and genes involved in chloroplast development (Sun et al. 2009). Two rice CMF proteins, named OsCMF8 (or grain number, plant height and heading date 7, Ghd7) and OsCMF1 (or OsCCT01), have been reported to modulate flowering time, plant height and grain number (Xue et al. 2008; Zhang et al. 2015).

In this study, we fused CIA2 and CIL fragments to either the β-glucuronidase (GUS) reporter sequence or to yeast-two hybrid system (Y2H) GAL4 activating domain (AD) and binding domain (BD) sequences. The GUS-fused constructs were transiently transformed into onion epidermal cells to identify functional NLS sequences in CIA2 and CIL. We then screened a Y2H Arabidopsis cDNA library to reveal putative CIA2-interacting proteins. We confirmed protein interactions by independent Y2H and biomolecular fluorescence complementation (BiFC) experiments. Based on our findings, we hypothesize that CIA2 and CIL not only regulate chloroplast function but also contribute to flower development.

## Results

### CIA2 has NLS at aa 62-65 and 291-308

Previous study has shown that CCT motifs serve as an NLS for many transcription factors (Kurup et al. 2000; Strayer et al. 2000; Robson et al. 2001). We used WoLF PSORT software (Horton et al. 2007) to predict the locations of NLS in CIA2, which identified NLS at aa 56-59 (RKPR), 62-65 (RKRP), 291-308 (K-rich) and 383-426 (CCT motif).

**Fig. 1:**
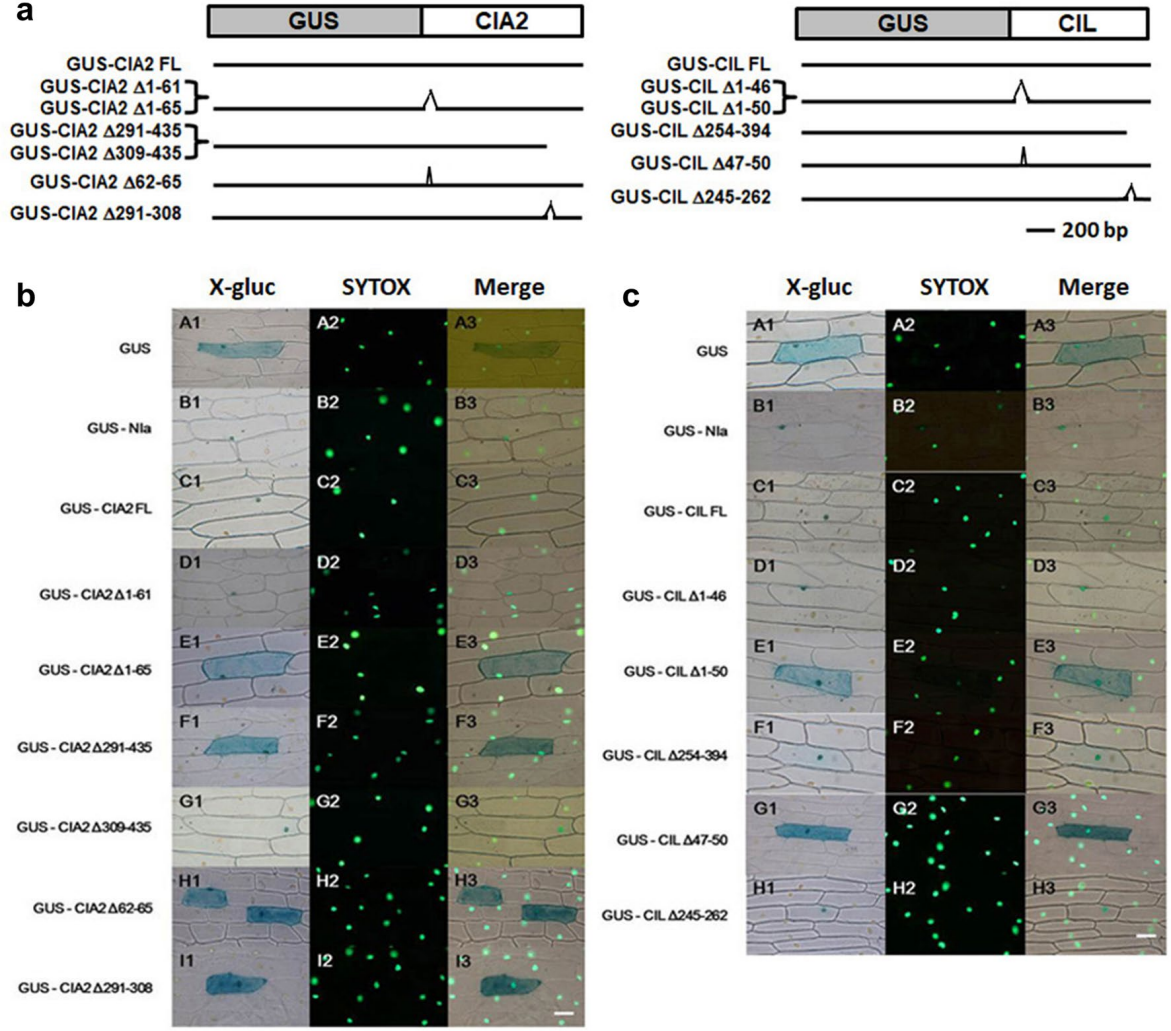
Cellular localizations of CIA2 and CIL fragments. Plasmids encoding various protein fragments, as labeled at left, were transformed into onion epidermal cells by means of particle bombardment. Samples were stained simultaneously with X-gluc and the nucleus-specific dye SYTOX. **a** Schematic diagram of the constructs used in this experiment. **b** CIA2-related constructs. **c** CIL-related constructs. Left row, visualization of X-gluc staining; middle row, visualization of SYTOX staining; right row, superimposition of X-gluc and SYTOX staining. Scale bars in **b** and **c** represent 50 μm

To establish if these NLS were functional, we fused full-length (FL) and various deletion fragments of CIA2 to the C terminus of the GUS coding sequence (Fig. 1a). Potyvirus nuclear inclusion protein (GUS-NIa) represented the positive control (Carrington et al. 1991). These chimeric constructs were driven by the CaMV 35S promoter and were transiently expressed in onion epidermal cells by microparticle bombardment. GUS-CIA2FL, GUS-CIA2Δ1-61 and GUS-CIA2Δ309-435 (Fig. 1b C1-3, D1-3 and G1-3) were all localized in the nucleus, demonstrating that aa 1-61 and 309-435 of CIA2 are not essential for protein entry into the nucleus. However, signal for GUS-CIA2Δ1-65, GUS-CIA2Δ291-435, GUS-CIA2Δ62-65, GUS-CIA2Δ291-308 (Fig. 1b E1-3, F1-3, H1-3 and I1-3) was evenly distributed throughout the cells, indicating that these deleted fragments might include functional NLS. Thus, CIA2 has two functional NLS at aa 62-65 and 291-308, so the CCT motif in CIA2 is not a functional NLS because that motif is localized at aa 383-426.

### CIL is a nuclear protein and its NLS is located at aa 47-50

We also employed WoLF PSORT to predict the subcellular localization of CIL and the sequence position of its NLS. The software predicted four NLS at aa 41-44 (RRPR), 47-50 (RKRP), 255-262 (K-rich) and 342-385 (CCT motif). Applying a similar strategy to that used for CIA2, we found that chimeric GUS-CIL FL, GUS-CILΔ1-46, GUS-CILΔ254-394 and GUS-CILΔ245-262 (Fig. 1c C1-3, D1-3, F1-3 and H1-3) all localized in the nucleus, suggesting that CIL aa 1-46 and 245-394 are not essential for nuclear entry. However, signal of both GUS-CILΔ1-50 and GUS-CIA2Δ47-50 (Fig. 1c E1-3 and G1-3) was evenly distributed in the cells, indicating that CIL has an NLS at aa 47-50. Thus, as for CIA2, the CCT motif in CIL (aa 341-384) is not a functional NLS.

### CIA2 interacts with other nuclear proteins

CIA2 may form complexes with other nuclear proteins to modulate physiological or developmental processes. To explore the biological functions of CIA2, we used a bait plasmid pGBKT7 containing fused GAL4 BD and 435-aa CIA2 fragments to screen an Y2H Arabidopsis cDNA library (Clontech Mate and Plate Library - Universal Arabidopsis) for putative CIA2-interacting proteins.

We identified 12 CIA2-interacting nuclear proteins from this assay, four of which represent nuclear proteins with unknown functions (Table 1). Notably, CIA2 is able to interact both with itself or CIL, indicating that CIA2 might form a homodimer or heterodimer to interact with other proteins. Three of the 12 identified proteins are nuclear factor Y subunit subdomains (i.e., NF-YB1, NF-YC1 and NF-YC9). Also known as CCAAT-box factors (CBF) or HEME activator proteins (HAP), NF-Y proteins have been independently identified from yeast, mammals, and plants, and are transcription factors and histone-folding proteins that are conserved across species (Mantovani 1999). NF-Y proteins comprise three major subunits—NF-YA (CBF-B or HAP2), NF-YB (CBF-A or HAP3), and NF-YC (CBF-C or HAP5)—that together can form heterotrimeric complexes and interact with other proteins to influence many aspects of plant life, such as seed development, flowering time regulation, primary root elongation, abscisic acid signaling, and drought resistance (Mantovani, 1999; Petroni et al. 2012; Zhao et al. 2016). Thus, through its interactions with NF-YB1, NF-YC1 or NF-YC9, CIA2 could participate in some of those biological processes.

**Table 1:** Putative CIA2-interacting proteins

Gene no. (AGI^a^)	Description	PSL^b^	Y2H^**c**^
AT1G08970	NUCLEAR FACTOR Y, Subunit C9 (NF-YC9); HEME ACTIVATED PROTEIN 5C (HAP5C)	N	**+**
AT1G59940	ARABIDOPSIS RESPONSE REGULATOR 3 (ARR3)	N	**+**
AT2G33390	Hypothetical Protein	N	
AT2G33400	FK506-BINDING NUCLEAR-LIKE PROTEIN	N	
AT2G38880	NUCLEAR FACTOR Y, Subunit B1 (NF-YB1); HEME ACTIVATED PROTEIN 3A (HAP3A)	N	**+**
AT3G07740	HOMOLOGY OF YEAST ADA2 2a (ADA2a)	N	
AT3G48590	NUCLEAR FACTOR Y, Subunit C1 (NF-YC1); HEME ACTIVATED PROTEIN 5A (HAP5A)	N	**+**
AT3G24650	ABSCISIC ACID INSENSITIVE 3 (ABI3)	N	**+**
AT4G16100	Putative Heat Shock Protein	N	
AT4G25990	CHLOROPLAST IMPORT APPARATUS 2-LIKE (CIL)	N	**+**
AT5G15840	CONSTANS (CO)	N	**+**
AT5G57180	CHLOROPLAST IMPORT APPARATUS 2 (CIA2)	N	**+**

^a^AGI, Arabidopsis Genome Initiative number

^b^PSL, Predicted subcellular localization based on SUBA3 database (Tanz et al. 2013). N, nucleus

^c^ +, subjected to Y2H assay

CO, another of the 12 interacting partners, is a 373-aa transcription factor that induces transcription of the *flowering locus T* (*FT*) gene, which controls the floral transition of shoot apical meristem (Abe et al. 2005; Corbesier et al. 2007; Jaeger and Wigge 2007). In Arabidopsis, CO forms a protein complex with NF-Y proteins. CO/NF-Y complexes bind to CO-response element (CORE) and the CCAAT-box of the *FT* promoter to increase *FT* expression and induce flowering (Wenkel et al. 2006; Tiwari et al. 2010). ABSCISIC ACID INSENSITIVE 3 (ABI3) also interacts with CO (Kurup et al. 2000), with the maize ABI3 orthologue VIVIPAROUS 1 (VP1, Finkelstein et al. 2002) playing essential roles in abscisic acid-mediated regulation of seed maturation, sensitivity to desiccation, and precocious germination (Ooms et al. 1993; Nambara et al. 1994; Parcy et al. 1994, 1997). However, manifestation of a late-flowering phenotype upon ectopic ABI3 expression and the early-flowering phenotype of the *abi3*-*4* mutant suggest that this protein is also involved in regulating flowering time (Kurup et al. 2000; Hong et al. 2019).

Moreover, ARABIDOPSIS RESPONSE REGULATOR 3 (ARR3) and ARR4 exhibit redundant functions in regulating signaling cascades in response to cytokinin and rhythmic expression of oscillator genes such as *circadian clock associated* 1 (*CCA1*) and *TOC1* (Kakimoto, 2003; Salomé et al. 2006). Taken together, the CIA2-interacting proteins ABI3, ARR3, CO, NF-YB1, NF-YC1 and NF-YC9 all seem to be at least partially involved in controlling flowering, strongly indicating a role for CIA2 in flower development.

### Confirmation of protein interactions among CIA2-interacting candidates

To confirm the interactions between CIA2, CIL and these six flowering regulators, we ligated different CIA2 and CIL fragments to pAS2-1 vector harboring GAL4 BD or the full-length (FL) proteins to pACT2 vector harboring GAL4 AD for Y2H assay. Plasmids were co-transformed into AH109 yeast cells and plated on medium with various concentrations of 3-AT. We further validated our findings by colony-lift filter assay to determine expression levels of β-galactosidase (LacZ) activity.

**Fig. 2:**
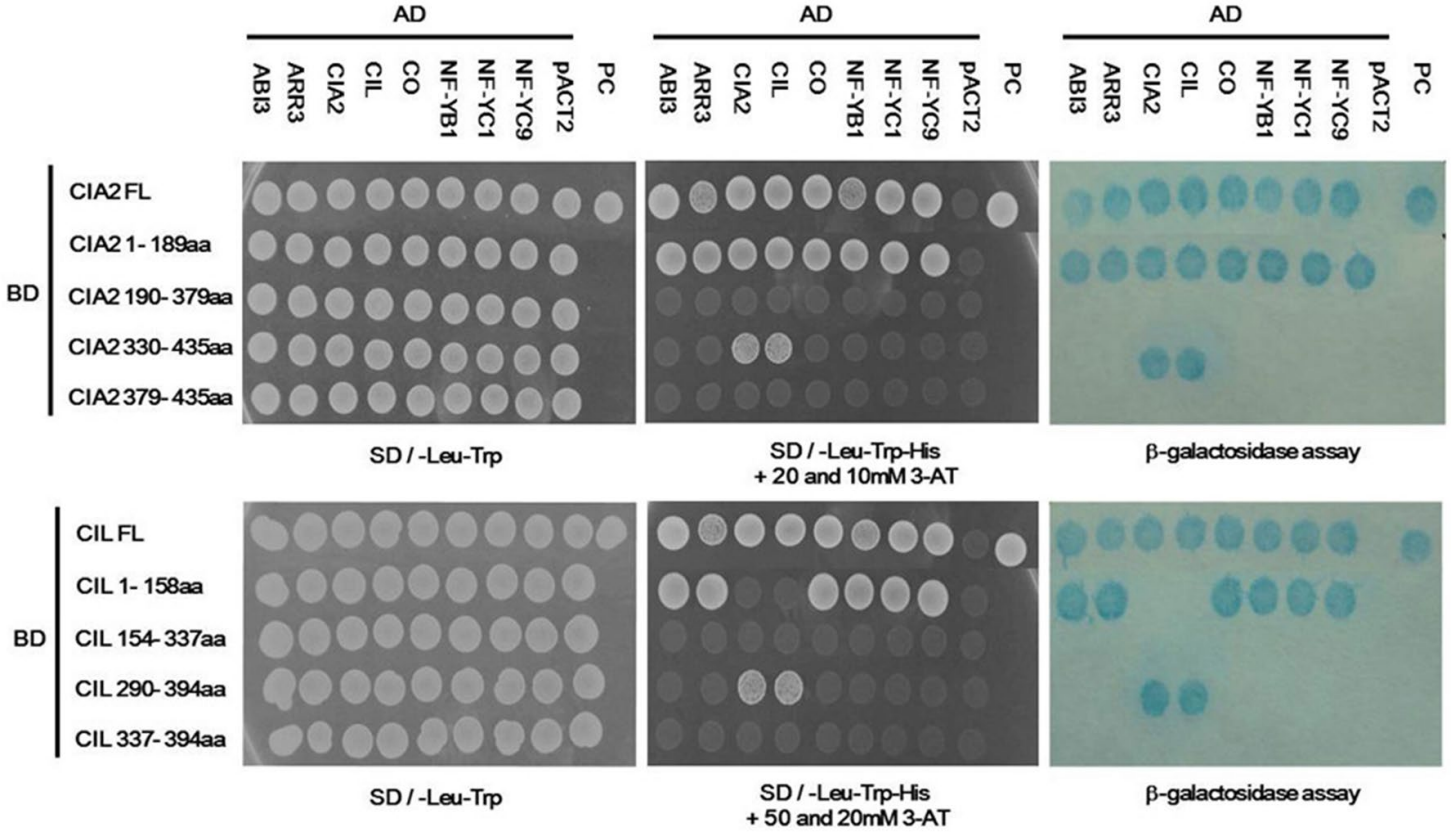
Interactions among CIA2 and CIL fragments and various proteins, as revealed by Y2H assay. Vectors pAS2-1 and pACT2 harbor GAL4 BD and GAL4 AD, respectively. The CIA2 and CIL fragments were ligated into pAS2-1 vector. Eight full-length CIA2-interacting protein candidates were ligated into pACT2 vector. Colonies were assayed for SD/-Leu-Trp autotrophy, SD/-Leu-Trp-Trp + 20 or 50 mM 3-AT autotrophy, and β-galactosidase activity. pACT2, negative control. PC, positive control containing pVA3-1 and pTD1-1

We found that only full-length CIA2 (CIA2FL) and the CIA2 1-189aa fragment interacted with all eight CIA2-interacting candidates (Fig. 2), showing that it is the N-terminal 1-189 fragment of CIA2 that interacts with other proteins. Notably, CIA2 could also interact with CIL and itself through the C-terminal 330-435 aa. However, the CCT motif (CIA2 379-435aa) alone was not sufficient for interaction with any candidate proteins. Similar to CIA2, CIL also interacted with the six flowering regulators via its N-terminal 1-156aa fragment. Interestingly, CIL only interacted with CIA2 and itself via the C-terminal 290-394aa fragment. Again, the CCT motif (CIL 337-394aa) alone was not sufficient to interact with any candidate proteins.

**Fig. 3:**
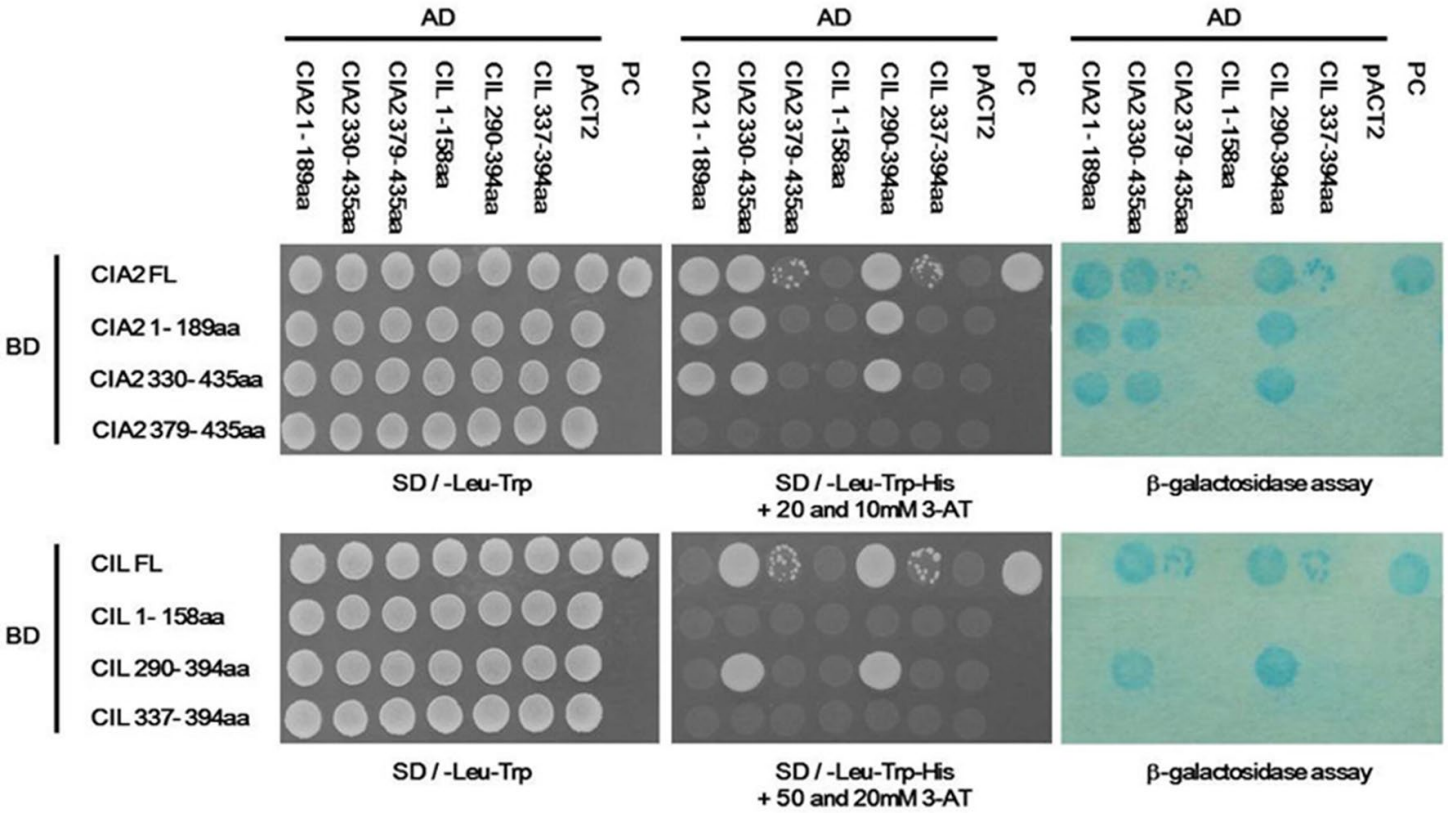
Domain swap analysis of CIA2 and CIL fragments, as determined by Y2H assay. The pAS2-1 and pACT2 vectors harbor GAL4 BD and GAL4 AD, respectively. The CIA2 and CIL fragments were ligated into pAS2-1 or pACT2 vectors. Colonies were assayed for SD/-Leu-Trp autotrophy, SD/-Leu-Trp-Trp + 20 or 50 mM 3-AT autotrophy, and β-galactosidase activity. pACT2, negative control. PC, positive control containing pVA3-1 and pTD1-1

To exclude bias from a plasmid effect in the divergent self- and cross-interaction results for CIA2 and CIL, we performed a domain swap analysis. We found that both the N-terminal CIA2 1-189aa and C-terminal CIA2 330-435aa fragments were mutually interactive (Fig. 3). However, the N-terminal CIA2 1-189aa fragment no longer interacted with full-length CIL when it was placed in an AD vector, indicating that CIL indeed specifically interacts with the C termini of both CIA2 and itself via its C-terminal 290-394 fragment.

**Fig. 4:**
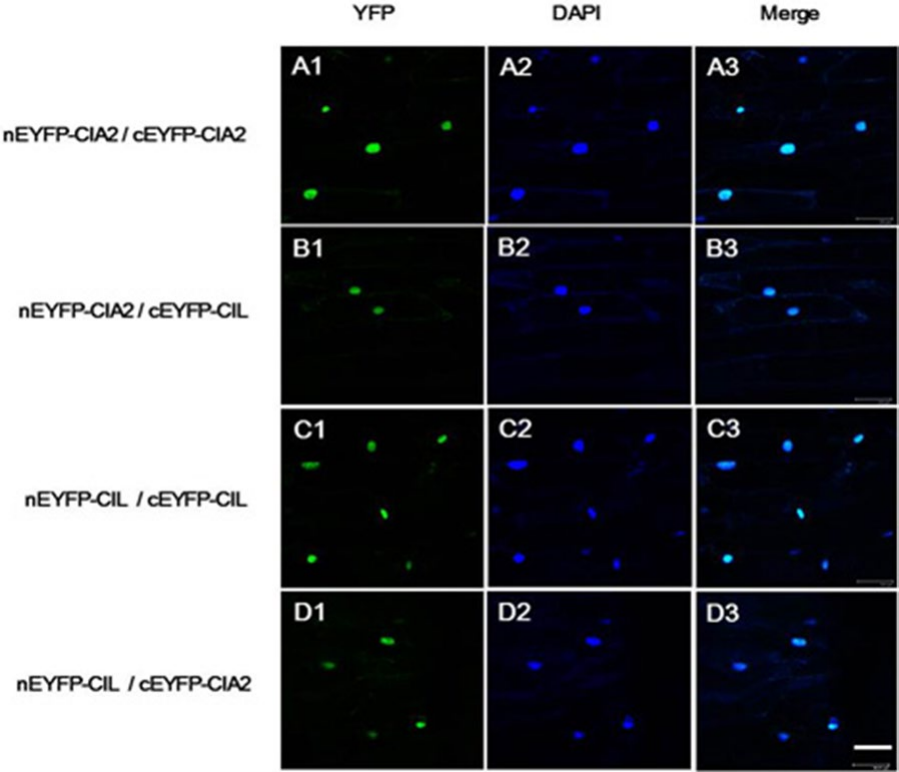
*In planta* interactions of CIA2 and CIL, as assessed by BiFC. Plasmids expressing nEYFP-CIA2, cEYFP-CIA2, nEYFP-CIL, or cYFP-CIL were co-transformed into onion epidermal cells by particle bombardment. A1 to D1, visualization of YFP signal. A2 to D2, visualization of DAPI staining. A3 to D3, superimposition of YFP and DAPI staining. YFP, yellow fluorescent protein. DAPI, nucleic acid dye. nEYFP, 1-173 aa fragment of the N terminus of eYFP; cEYFP, 174-328 aa fragment of the C terminus of eYFP. Scale bar represents 100 μm

Next, we used BiFC to establish if CIA2 and CIL interact *in planta*. Full-length CIA2 and CIL protein were separately fused behind the N or C terminus of enhanced yellow fluorescent protein (nEYFP or cEYFP), and transiently expressed in onion epidermal cells by particle bombardment (Waadt et al. 2008), and then observed by fluorescence microscopy. The results demonstrate that both CIA2 and CIL mutually interact in the nucleus of onion epidermal cells (Fig. 4).

### CIA2 and CIL interact with the CCT motif of CO

**Fig. 5:**
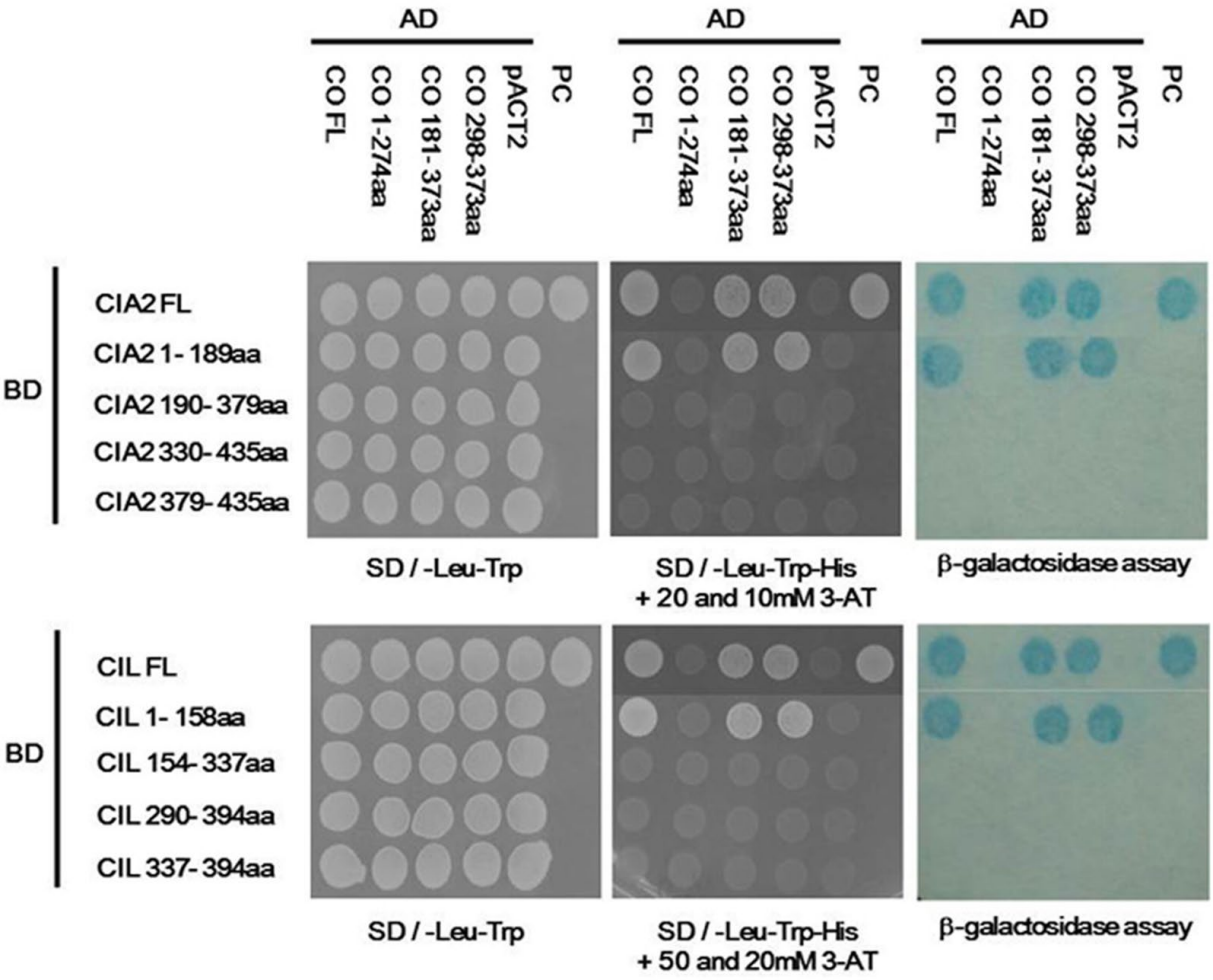
Interactions between CIA2/CIL and CO, as assessed by Y2H assay. Vectors pAS2-1 and pACT2 harbor GAL4 BD and GAL4 AD, respectively. The CIA2 and CIL fragments were ligated into pAS2-1 vector. The full-length CO fragment was ligated into pACT2 vector. Colonies were assayed for SD/-Leu-Trp autotrophy, SD/-Leu-Trp-Trp + 20 or 50 mM 3-AT autotrophy, and β-galactosidase activity. pACT2, negative control. PC, positive control containing pVA3-1 and pTD1-1

CO utilizes its C-terminal CCT motif to interact with the NF-YB and NF-YC subunits, with those interactions allowing CO/NF-Y complex to bind to the *FT* promoter to increase transcript yield of *FT*, thereby promoting flowering (Wenkel et al. 2006; Tiwari et al. 2010). However, alternative splicing of *CO* transcript generates a shorter 274-aa CO protein lacking the CCT motif at the C terminus. This truncated CO protein interacts with full-length 373-aa CO at the N-terminus, causing degradation of the 373-aa CO spliced variant to delay flowering time (Gil et al. 2017). Determining how CIA2/CIL and CO interact would help establish if and how CIA2/CIL is involved in flowering regulation. We used Y2H assay to investigate this topic. We found that CIA2FL and CIA2 1-189aa interacted with the COFL, CO 181-373aa and CO 298-373aa fragments (each of which harbors the CCT motif), but the CIA 190-379aa, CIA2 330-435aa and CIA2 379-435aa did not (Fig. 5). We observed similar results for CIL fragments (Fig. 5). Together, these data show that it is the N-terminal regions of CIA2/CIL that interact with the C-terminal CCT motif of CO.

### Evolutionary relationships of CIA2 and CIL homologues

To investigate the evolutionary relationships between CIA2 and CIL and further understand their physiological mechanisms, we conducted bioinformatics analysis on homologous proteins. We anticipated that proteins playing an important regulatory role in plant cells would exhibit conserved motifs across species. Since we had found that it is the N-termini of CIA2 and CIL that interact with the six flowering regulators (Table 1 and Fig. 2), we conducted a BLAST analysis of the N-terminal 200-aa sequences of Arabidopsis CIA2 and CIL to extract homologous sequences from the Gramene (http://www.gramene.org) and NCBI (National Center for Biotechnology Information; http://www.ncbi.nlm.nih.gov) databases (Altschul et al. 1997). We obtained 280 protein sequences using default settings, revealing that CIA2 and CIL homologues are widely present across the plant kingdom. In Additional file 1: Table S1, we present summary data on 70 angiosperms (such as plants of the Cruciferae, Solanaceae, Fabaceae and Poaceae families) and 2 gymnosperms (*Ginkgo biloba* and the early flowering *Amborella trichopoda*) for which full-length sequences were available.

**Fig. 6:**
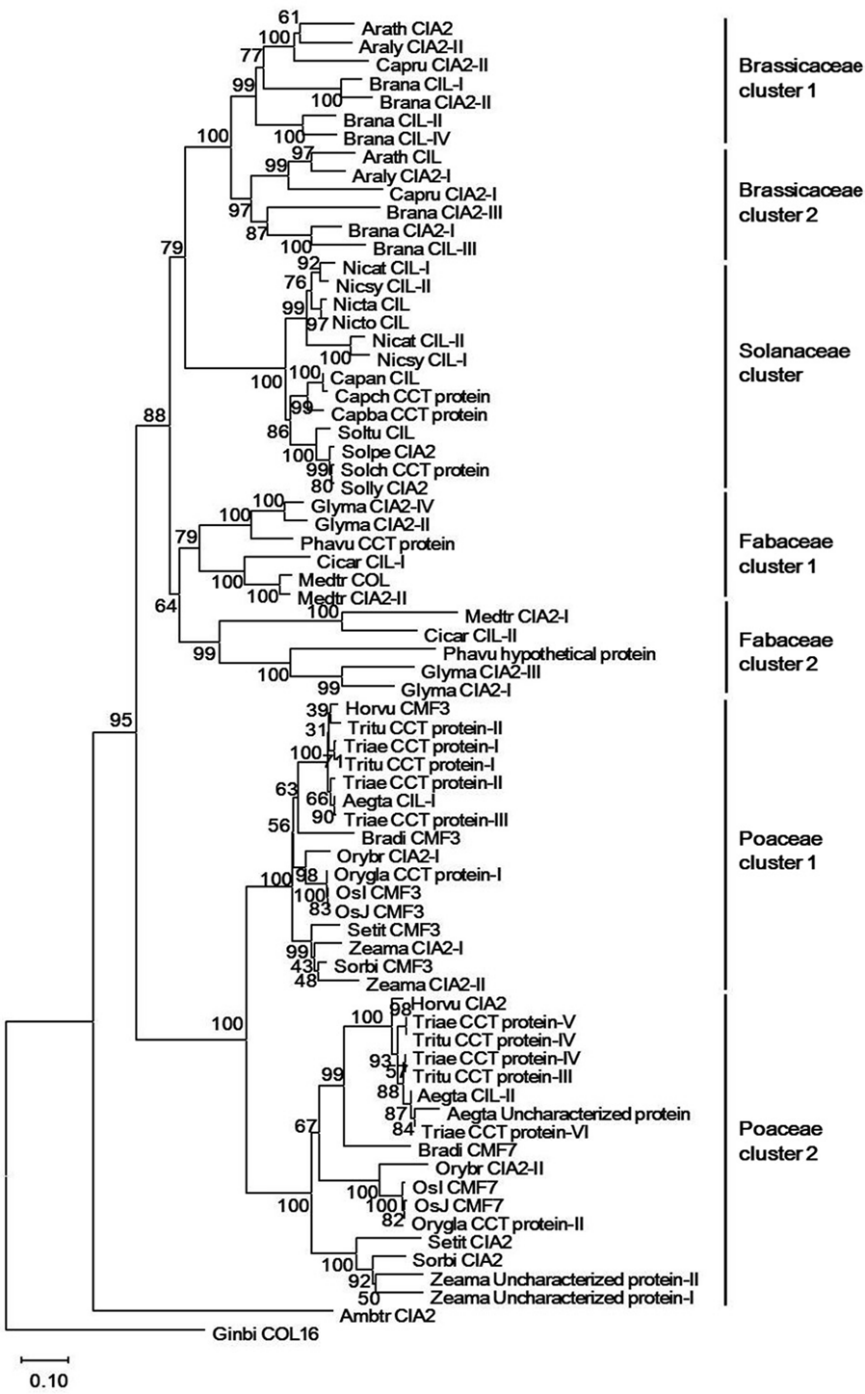
Phylogenetic tree of 72 CIA2 and CIL protein homologs, generated by the MEGA-X software using a Neighbor-Joining approach with bootstrapping. Numbers at the roots of branches represent credible values obtained by performing 1000 bootstraps. Branch lengths have been drawn according to evolutionary distance, and the respective scale is shown in the lower left corner. Sequence details are provided in Supplementary Table 1

Phylogenetic analyses of N-terminal 200-aa sequences from these 72 plants using MEGA-X software (Kumar et al. 2018) revealed that CIA2 and CIL evolved from the same ancestral protein (Fig. 6). For each of the Brassicaceae, Fabaceae and Poaceae families, we observed two clusters of generally intermixed CIA2 and CIL homologues, but only one cluster for solanaceous plants. The resulting phylogeny demonstrates that the ancestral gene of CIA2/CIL has convergently replicated into two functional sequences and subsequently diverged during the evolution of true flowering plants. Such an evolutionary process has occurred independently in various plant families (Fitter et al. 2002).

### Significance of the N-terminal CC1 motif of CIA2/CIL for interactions with flowering regulators

Apart from the CCT motif, previous bioinformatics analysis using the MEME software (Bailey et al. 2009) identified five additional conserved motifs in CIA2 and CIL (named CC1 ~ 5, Additional file 2: Fig. S1). These motifs in CIA2 are located at aa 45-86, 162-188, 252-262, 334-359 and 364-381 for CC1 to CC5, respectively, whereas in CIL they are located at aa 30-71, 137-163, 205-215, 290-315 and 322-339.

**Fig. 7:**
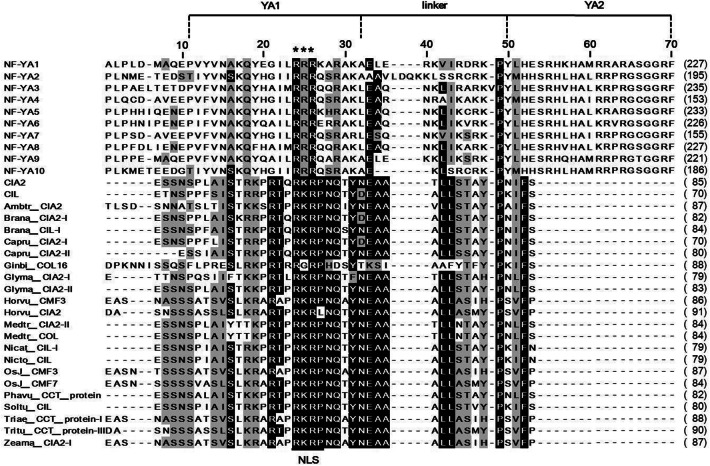
Sequence alignment (using MEGA-X software) of a conserved region of 10 Arabidopsis NF-YA proteins and 23 CIA2/CIL homologous proteins. Numbers in brackets at the end of each sequence indicate the corresponding aa number of each protein. Numbers above the alignment represent amino acid position (in the alignment). Black and gray backgrounds represent conserved or similar residues, respectively. Residues marked with an asterisk are important in yeast NF-YA protein. The YA1/linker/YA2 subdomain region is demarcated based on Romier et al. (2003). The location of the NLS sequence in the CC1 motifs of homologous proteins is shown below the alignment

Interestingly, the CC1 motif covers the NLS we identified in CIA2 and CIL (Fig. 1). Using the MUSCLE software in MEGA-X (Edgar, 2004a and 2004b; Kumar et al. 2018), we found that the amino acid sequence of the CC1 motif is similar to highly-conserved subdomain YA1 of NF-YA subunits (Fig. 7). The YA1 subdomain interacts with NF-YB/NF-YC dimer (Romier et al. 2003), and three of its arginine residues (R24, R25 and R26) modulate NF-Y complex assembly. R24K or R24-26A mutation of Arabidopsis NF-YA proteins (also named HAP2) resulted in lack of HAP complex formation and impeded DNA binding (Mantovani, 1999). Moreover, R25G and R26P mutation of HAP2 also abolished HAP complex formation and resulted in loss of DNA binding, but the R25K mutation did not affect HAP2 function (Xing et al. 1994). Since residues 24-26 of the YA1 subdomain are almost identical to the NLS of the CC1 motif of CIA2/CIL (Fig. 7), we speculate that CIA2 and CIL interact with CO and NF-Y proteins via their N-terminal CC1 motifs.

## Discussion

Proteins harboring CCT motifs are plant-specific transcription factors. The highly conserved CCT motif principally functions as an NLS and enables protein–protein interactions in many such transcription factors. For example, the sole NLS of the photoperiodic flowering regulator CO is a CCT motif (Robson et al. 2001). In this study, we demonstrate that both CIA2 and CIL have NLS localized at their N termini instead of in the CCT motif (Fig. 1) and, furthermore, that the CCT motif is not sufficient to mediate CIA2 and CIL protein interactions (Figs. 3 and 5).

The RKRP sequence located at aa 62-65 and aa 47-50 of CIA2 and CIL, respectively, is similar to the NLS of Simian Virus 40 large T antigen type (Kalderon et al. 1984). The Arabidopsis GOLDEN 2-like proteins, GLK1 and GLK2, that regulate chloroplast development also contain this NLS-type sequence (Fitter et al. 2002). However, CIA2 has another NLS located at aa 291–308. Both of these NLS are required to ensure protein entry into the nucleus, clarifying why the Arabidosis *cia2* mutant is defective in chloroplast development. The *cia2* mutant develops a truncated 256-aa CIA2 protein that lacks this latter NLS, so fails to enter the nucleus. Consequently, expression of genes in the nucleus related to chloroplast development are decreased. We postulate that CIA2 possesses two NLS to increase nuclear-entry efficiency, given that the GATA transcription factor AreA in *Aspergillus nidulans* exhibts greater efficiency in entering the nucleus depending on the number of NLS present (Hunter et al. 2014).

Recent study has indicated that *Hordeum vulgare* (barley) ALBOSTRIANS protein (HvAST, also known as HvCMF7 or HvCIA2) is a chloroplast protein rather than a nuclear transcription factor (Li et al. 2019). These authors claimed that the CIA2 homologous protein has different subcellular localization in barley. Nonetheless, our previous publications and the current study strongly demonstrate that Arabidopsis CIA2 and CIL are nuclear proteins (Figs. 1 and 4; Sun et al. 2001, 2009).

The cytosolic heterodimer of NF-YB and NF-YC enters the nucleus to form an NF-Y complex with NF-YA, which then binds to the CCAAT element of gene promoters (Romier et al. 2003). The CCT motif of CO is very similar to the YA2 subdomain of NF-YA subunits, so CO can replace NF-YA to form a large transcriptional complex with NF-YB1 and NF-YC1 that regulates *FT* expression (Wenkel et al. 2006). CO also forms transcriptional complexes with other NF-YB (B2 or B3) and NF-YC (C3, C4 or C9) subunits, and then binds to the CORE and CCAAT elements of the *FT* promoter to enhance flowering (Kumimoto et al. 2010; Xu et al. 2016). Overexpression of NF-YC1 or parallel overexpression of both CO and NF-YB1 in phloem causes early flowering (Wenkel et al. 2006). Since CIA2 and CIL interact with CO, NF-YB1, NF-YC1 and NF-YC9 via their CC1 motifs (Fig. 2), CIA2 and CIL may contribute to FT-related flowering control. Overexpression of CIA2 and CIL, together with CO, in transgenic plants and assessment of any resulting alterations in flowering time will provide greater clarity on the mechanism by which CIA2 and CIL are involved in FT-related flowering. Alternatively, a genetic approach using single, double, or even triple mutants could be deployed.

Our Y2H screening did not provide any evidence for an interaction between NF-YA proteins and either CIA2 or CIL (Table 1). However, since CO may act analogously to NF-YA in NF-Y complexes (Wenkel et al. 2006), and there is high sequence similarity between the NF-YA1 subdomain and linker and CIA2 and CIL proteins (Fig. 7), CIA2/CIL might substitute for NF-YA proteins to interact with CO and NF-YB1/NF-YCs (C1 and C9).

NF-Y subunits are differentially expressed in various tissues during developmental stages (Gusmaroli et al. 2001, 2002) or in response to environmental change (Des Marais et al. 2012). For example, NF-YC9 is associated with chlorophyll biosynthesis in Arabidopsis (Warpeha et al. 2007), NF-YB1 regulates drought stress responses independently of ABA signaling, and NF-YC1 positively regulates freezing responses (Nelson et al. 2007; Shi et al. 2014). Therefore, further analyses of the interactions of CIA2 and CIL with other proteins and themselves may reveal novel functions.

A previous Y2H study demonstrated that ABI3 (which we have identified here as interacting with CIA2/CIL, Table 1) suppresses CO function by blocking the CCT domain of CO (Kurup et al. 2000). The *abi3* mutant exhibits an early flowering phenotype (Kurup et al. 2000). We found that both CIA2 and CIL interact with ABI3 and CO via their respective N-termini (Fig. 2), so CIA2 and CIL may participate in regulating ABI3 and CO interaction, though this possibility requires experimental analysis.

Our phylogenetic analysis indicates that CIA2 and CIL arose from duplication of the same ancestral gene, which ultimately diverged in different plant families due to functional requirements (Fig. 6). Our previous study demonstrated that *CIA2* and *CIL* are expressed in leaves, flowers and fruits, but not in roots (Sun et al. 2001). Moreover, expression of *CIL* is moderately increased in the *cia2* mutant over wild-type, suggesting that CIL might be functionally redundant to CIA2 (Sun et al. 2001). Here, we found that both CIA2 and CIL can interact with CO and NF-YB1/NF-YCs (C1 and C9) via their N-terminal CC1 motif, suggesting that they might be involved in flowering regulation. Arabidopsis CIA2 has a higher sequence identity than CIL to the ancestral proteins in *Ginkgo biloba* and *Amborella trichopoda* (Additional file 1: Table S1), meaning that *CIL* most likely arose from gene duplication of an ancestral-like *CIA2* gene. More in-depth study of CIA2 and CIL will help establish if these two genes have different functions.

CO and COL proteins interact with NF-YBs/NF-YCs and ABI3 via their C-terminal CCT motifs (Kurup et al. 2000; Wenkel et al. 2006; Xu et al. 2016), but CIA2 and CIL interact with CO, NF-YB1/NF-YCs (C1 and C9) and ABI3 through their N-terminal CC1 motif (Fig. 2 and 5). We postulate that any amino acid substitution in the CC1 domains of CIA2 or CIL will render the resulting mutant proteins incapable of interacting with other proteins. Wenkel et al. (2006) reported that four arginine residues (R3, R17, R35, and R37) and 1 phenylalanine residue (F42) of the CCT motif are indispensable to the protein–protein interaction and DNA-binding abilities of CO and COL proteins. Indeed, an alignment of 20 CIA2, CIL, CO, COL and TOC1 proteins revealed that all five of these residues are conserved (Additional file 3: Fig. S2). However, several residues in the CCT motifs of CIA2 and CIL are clearly divergent to those of the other proteins. For instance, CIA2 and CIL have a serine (S) at residue 6, whereas all other proteins have either arginine (R) or alanine (A) at the same location (Additional file 3: Fig. S2). We plan to use site-directed mutagenesis to generate S6R or S6A mutations in the CCT motifs of CIA2 and CIL, allowing us to assess changes in interactions among CIA2, CIL, and flowering-control proteins.

## Conclusions

Arabidopsis CIA2 and CIL are CMF-class proteins, exhibiting a domain of unknown function at the N terminus and a CCT motif in the C terminus. In this study, we show that the N termini of CIA2 and CIL have a conserved CC1 motif, which is highly similar to the YA1 subdomain of NF-YA proteins. This CC1 motif not only serves as an NLS for CIA and CIL, but also specifically interacts with flowering regulators, such as ABI3, ARR3, CO, NF-YB1, NF-YC1 and NF-YC9. Based on these findings and those of our previously published studies, we propose that apart from regulating chloroplast function, Arabidopsis CIA2 and CIL can interact with ABI3, ARR3, CO and NF-Y complex to modulate flower development.

## Methods

### Subcellular localization

The nuclear localization assay was conducted as described previously (Sun et al. 2001). Polymerase chain reaction (PCR)-amplified CIA2 or CIL fragments were preferentially digested with Bam HI and Xba I, and cloned into Bgl II- and Xba I-digested plasmid pRG/NIa1-76 (Carrington et al. 1991) to replace the NIa1-76 fragment. The CIA2Δ62-65 and CIA2Δ291-308 fragments were subcloned by Mun I (Mfe I) and Kpn I digestion. The CILΔ47-50 and CILΔ245-262 fragments were subcloned by Hpa I and Alf II digestion. The resulting fusion constructs were transiently expressed in onion epidermal cells by microparticle bombardment using the Biolistic PDS-1000/He Particle Delivery System (Bio-RAD) as described (Varagona et al. 1992). SYTOX Green Stain (Molecular Probes) is a green-fluorescing nuclear dye. SYTOX-stained nuclei were observed using Leica TCS SP2 confocal microscope in the fluoresce channel, and localization of 5-bromo-4-chloro-3-indolyl-β-glucuronic acid (X-gluc) staining was observed using the same microscope in the transmission channel.

### Y2H screening of an Arabidopsis cDNA library

A normalized library made from 11 Arabidopsis tissues (Mate and Plate Library, Universal Arabidopsis, Clontech) was transformed into yeast strain Y187, and then the Matchmaker Gold Yeast Two-Hybrid System protocol (Clontech) was applied. The bait pGBKT7 vector containing GAL4 BD and full-length CIA2 (ligated with Nco I and Bam HI) was transformed into the AH109 yeast strain (Clontech). Strain combinations were mixed at 30 °C for 24 h, and the resulting zygotes were plated directly on SD/–Leu–Trp–His growth medium supplemented with 5 mM 3-Amino-1,2,4-triazole (3-AT). After incubating at 30 °C for 6 days, colonies exhibiting a growth surface diameter of > 1 mm were transferred on SD/-Leu-Trp-His containing 10 mM 3-AT. Colonies were further assessed by β-galactosidase (β-gal) activity assay. Plasmids from blue yeast colonies that survived stringent selection were extracted and sequenced using the GAL4 5′AD or T7 primers. Translated cDNA sequences in-frame to the GAL4-AD sequence were selected and further analyzed.

### Y2H assay

Our Y2H assay protocol follows the Matchmaker Yeast Two-Hybrid System 2 (Clontech). The pAS2-1 and pACT2 vectors contain GAL4 BD and GAL4 AD, respectively. The coding sequences of ABI3, ARR3, CO, CIA2, CIL and NF-Y proteins were amplified by PCR using gene-specific primers (Table 2) from cDNA templates of 18 or 28 day-old wild-type Arabidopsis plants. PCR fragments were digested at specific restriction sites: Bam HI and Sal I (for ABI3), Nco I and Bam HI (for ARR3), Sma I and Bam HI (for CO fragments), Nco I and Sma I (for CIA2), Nco I and Sma I (for CIL), Nco I and Eco RI (NF-YB1), Nco I and Eco RI (for NF-YC1), and Bam HI and SalI (for NF-YC9). The digested fragments were ligated to pAS2-1 and pACT2. The resulting plasmids were sequence-verified. These plasmids were transformed into the AH109 yeast strain and protein interactions were verified by growth in SD/–Leu–Trp–His growth medium supplemented with various concentrations of 3-AT. After incubation at 30 °C for 6 ~ 9 days, expression of the reporter gene *lacZ* was further confirmed by colony-lift filter assay. We used o-nitrophenyl β-D-galactopyranoside (ONPG) as substrate to quantify LacZ activity, following an experimental procedure described previously (Miller 1972).

**Table 2:** Gene-specific primer sequences of CIA2-interacting proteins used in this study

Protein (Restriction enzyme)	Forward primers (5′ to 3′)^a^	Reverse primers (5′ to 3′)
ABI3 (BamHI and SalI)	CGGGATCCCGATGAAAAGCTTGCATGTGGC	GCGTCGACTCATTTAACAGTTTGAGAAGTTGG
ARR3 (NcoI and BamHI)	CGCCATGGCCAAAGACGGTGGCGTTTC	CGGATCCCTAAGCTAATCCGGGACTCCTC
CO (SmaI and BamHI)	CGCCCGGGGATGTTGAAACAAGAGAGTAACGAC	GCGGATCCTCAGAATGAAGGAACAATCCCATATC
CIA2 (NcoI and SmaI)	CGCCATGGAGATGTCGGCGTGTTTAAGCAGCG	CGCCCGGGTTATCTTTGTCCACTTGGAGTG
CIL (NcoI and SmaI)	CGCCATGGAGATGTCCTCTTGTGCCTATAGC	CGCCCGGGTAATCGTAGACCACTTAAATTCC
NF-YB1 (NcoI and EcoRI)	CATGCCATGGCGGATACGCCTTCGAG	GGAATTCTTACCAGCTCGGCATTTCTTCA
NF-YC1 (NcoI and EcoRI)	CATGCCATGGATACCAACAACCAGCAAC	GGAATTCTTAACCTTGGCCGTCGAG
NF-YC9 (BamHI and SalI)	CGGGATCCGAATGGATCAACAAGACCATGG	GCGTCGACTAATTTTCCTGGTCAGGTTGG

^a^The underlined sequences are additional restriction sites

### Bimolecular fluorescence complementation (BiFC) assay

Full-length CIA2 and CIL were PCR-amplified and inserted into the vectors pVYNE(R) or pVYCE(R) (Waadt et al. 2008), respectively. BiFC constructs were sequence-verified and then transiently expressed in onion epidermal cells by microparticle bombardment using the Biolistic PDS-1000/He Particle Delivery System (Bio-RAD) as described previously (Varagona et al. 1992). The two plasmids (1.25 μg of each) in a 1:1 molar ratio were mixed well. Signals of 4′,6-diamidino-2-phenylindole (DAPI)-stained nuclei and enhanced yellow fluorescent protein (eYFP) were detected using a Leica TCS SP2 confocal microscope. Excitation of the UV laser was used to observe DAPI fluorescence (455-465 nm), and the Ar/KrAr laser (488 nm) was used to visuzlize eYFP fluorescence (525-545 nm).

### Phylogenetic analyses

The N-terminal 200-aa sequences of Arabidopsis CIA2 and CIL were subjected to BLAST analysis for homologous sequences against the Gramene (http://www.gramene.org) and NCBI (National Center for Biotechnology Information; http://www.ncbi.nlm.nih.gov) (Altschul et al. 1997) databases. MUSCLE software with default settings was used to compare the full-length sequences (Edgar 2004a and b). We used MEGA-X software to draw and analyze a phylogenetic tree based on a Neighbor-Joining approach and for bootstrapping (Kumar et al. 2018). MEME software (Bailey et al. 2009) was used to identify conserved motifs in full-length sequences of homologous proteins.

## Supplementary information

**Additional file 1: Table S1.** List of CIA2 and CIL homologous proteins.

**Additional file 2: Figure S1.** CC1 ~ 5 motifs conserved between CIA2 and CIL proteins, as predicted by MEME suite.

**Additional file 3: Figure S2.** Comparison of CCT motifs of Arabidopsis CIA2, CIL, CO, COL, and TOC1 proteins.

## Abbreviations

ABI3: ABSCISIC ACID INSENSITIVE 3
CCT: CONTANS (CO), CO-LIKE (COL), and TIMING OF CAB EXPRESSION 1 (TOC1)
CIA2: CHLOROPLAST IMPORT APPARATUS 2
CIL: CIA2-LIKE
CPN: Chaperonin
cpRP: Chloroplast ribosomal protein
GUS: β-Glucuronidase
NF-Y: Nuclear Factor Y
NLS: Nuclear localization signal
Toc: Translocon at the outer envelope membrane of the chloroplast
X-gluc: 5-bromo-4-chloro-3-indolyl-β-glucuronic acid
Y2H: Yeast two-hybrid
