# Supplementary material for: Sequence analysis and protein interactions of Arabidopsis CIA2 and CIL proteins
Source: Bot Stud. 2020 Jun 18;61:20. doi: 10.1186/s40529-020-00297-z (PMC7303255; doi:10.1186/s40529-020-00297-z)
Supplement: Supplementary file 1 — Additional file 1: Table S1. List of CIA2 and CIL homologous proteins. [file 40529_2020_297_MOESM1_ESM.pdf]

Supplemental Data

Table S1. List of CIA2 and CIL homologous proteins.

The N-terminal 1-200 amino acid sequences of Arabidopsis CIA2 and CIL were subjected to BLAST analysis to identify homologous proteins in the NCBI and Gramene databases. Information on 72 full-length CIA2 and CIL homologues is summarized below.

Protein name[a]	Gene symbol / Gene locus[bc]	Amino acid	Species	Sequence ID	Description[d]	Percentage identity to CIA2 FL (%)[e]	E-value[e]	Percentage identity to CIL FL (%)[e]	E-value[e]
Aegta_CIL-I	LOC109759413 AET6Gv20305800[c]	494	*Aegilops tauschii subsp. tauschii*	XP_020173826.1	CCT(440-483aa)	32.15	2.00E-47	31.39	9.00E-49
Aegta_CIL-II	LOC109734132	457	*Aegilops tauschii subsp. tauschii*	XP_020148948.1	CCT(401-445aa)	27.4	4.00E-23	28.64	3.00E-25
Aegta_Uncharacterized protein	AET7Gv21020300[c]	267	*Aegilops tauschii subsp. tauschii*	UniProtKB-M8BAB9	-	39.39	6.00E-11	38.95	2.00E-09
Ambtr_CIA2	LOC18440680[b] AMTR_s00025p00156240[c]	436	*Amborella trichopoda*	XP_006850881.1	CCT(382-425aa)	32.53	3.00E-44	32.39	8.00E-43
Araly_CIA2-I	LOC9305708[b]	392	*Arabidopsis lyrata*	XP_002867585.1	CCT(339-382aa)	55.48	2.00E-132	85.71	0
Araly_CIA2-II	LOC9302264[b]	438	*Arabidopsis lyrata*	XP_020866733.1	CCT(386-429aa)	87.97	0	56.36	1.00E-131
CIL (CMF9[a])	AT4G25990[b]	394	*Arabidopsis thaliana*	NP_567737.1	CCT(341-384aa)	55.98	1.00E-129	100	0
CIA2 (CMF14[a])	AT5G57180[b]	435	*Arabidopsis thaliana*	NP_568852.2	CCT(383-426aa)	100	0	54.5	2.00E-129
Bradi_CMF7[a]	LOC100822180[b] BRADI_1g34060[c]	448	*Brachypodium distachyon*	XP_003560511.1	CCT(398-436aa)	40	4.00E-10	28.5	5.00E-26
Bradi_CMF3[a]	LOC100822550[b] BRADI_3g03770[c]	459	*Brachypodium distachyon*	XP_014756479.1	CCT(416-452aa)	32.48	5.00E-38	33	3.00E-38
Brana_CIA2-I	LOC106444616[b] BnaA01g15160D[c]	382	*Brassica napus*	XP_013741528.1	CCT(327-372aa)	53.29	3.00E-130	64.25	1.00E-164
Brana_CIL-I	LOC106388956[b] BnaA02g08300D[c]	392	*Brassica napus*	XP_013684589.1	CCT(340-383aa)	64.03	9.00E-164	50.99	2.00E-113
Brana_CIL-II	LOC106371508[b] BnaA10g11220D[c]	397	*Brassica napus*	XP_013667045.1	CCT(345-388aa)	62.5	1.00E-150	56.18	5.00E-114
Brana_CIL-III	LOC106376068[b] BnaC01g17920D[c]	391	*Brassica napus*	XP_013671573.1	CCT(336-381aa)	53.02	5.00E-131	64.51	3.00E-163
Brana_CIA2-II	LOC106377535[b]	386	*Brassica napus*	XP_013673245.1	CCT(334-377aa)	60.22	5.00E-153	50.37	7.00E-109

	BnaC02g11770D[c]								
Brana_CIA2-III	LOC106409124[b] BnaC07g39980D[c]	397	*Brassica napus*	XP_013705259.1	CCT(344-389aa)	50.66	1.00E-96	63.1	5.00E-127
Brana_CIL-IV	LOC106418421[b] BnaC09g53340D[c]	398	*Brassica napus*	XP_013714579.1	CCT(346-389aa)	63.98	3.00E-159	56.61	7.00E-121
Capru_CIA2-I	LOC17879835	392	*Capsella rubella*	XP_006283882.1	CCT(339-382aa)	53.54	1.00E-117	78.3	0
Capru_CIA2-II	LOC17876953	436	*Capsella rubella*	XP_006280489.1	CCT(384-427aa)	71.71	0	51.05	8.00E-115
Capan_CIL	LOC107877814	441	*Capsicum annuum*	XP_016580048.1	CCT(375-417aa)	43.76	4.00E-86	42.4	9.00E-79
Capba_CCT protein	CQW23_16970	415	*Capsicum baccatum*	PHT42945.1	CCT(364-407aa)	43.74	6.00E-88	43.44	2.00E-80
Capch_CCT protein	BC332_18791	442	*Capsicum chinense*	PHU11861.1	CCT(376-434aa)	43.76	9.00E-87	42.65	6.00E-79
Cicar_CIL-I	LOC101508698	400	*Cicer arietinum*	XP_004507897.1	CCT(354-396aa)	46.56	2.00E-91	42.33	2.00E-78
Cicar_CIL-II	LOC101511273	303	*Cicer arietinum*	XP_004487994.2	-	32.34	2.00E-30	30.77	8.00E-25
Ginbi_COL16	N/A	436	*Ginkgo biloba*	ASW41094.1	CCT(381-424aa)	44.62	1.00E-23	42.64	6.00E-22
Glyma_CIA2-I	LOC100783619 GLYMA_09G059800[c]	374	*Glycine max*	XP_006586995.1	CCT(344-373aa)	34.18	2.00E-46	35.75	3.00E-40
Glyma_CIA2-II	LOC100784788 GLYMA_13G100700[c]	399	*Glycine max*	XP_003543009.1	CCT(349-391aa)	43.58	2.00E-76	44.16	6.00E-78
Glyma_CIA2-III	LOC102670319 GLYMA_15G166500[c]	395	*Glycine max*	XP_006597800.1	-	34.34	4.00E-46	32.64	2.00E-36
Glyma_CIA2-IV	LOC100810303 GLYMA_17G059100[c]	398	*Glycine max*	XP_003549301.1	CCT(347-389aa)	45.11	5.00E-79	45.29	5.00E-77
Horvu_CMF3[a]	HORVU6Hr1G021460[c]	480	*Hordeum vulgare subsp. Vulgare*	UniProtKB-A0A287TJQ3	CCT(436-478aa)	32.34	1.00E-44	32.44	7.00E-47
Horvu_CIA2 (Horvu_CMF7[a])	HORVU7Hr1G097630[c]	459	*Hordeum vulgare subsp. Vulgare*	SNU42600.1	CCT(403-448aa)	29.59	2.00E-23	27.89	2.00E-25
Medtr_CIA2-I	LOC11443621 MTR_2g038010[c]	313	*Medicago truncatula*	XP_003595074.1	-	32.04	6.00E-31	30.32	6.00E-26
Medtr_CIA2-II	LOC11441754 MTR_4g127420[c]	379	*Medicago truncatula*	XP_003610054.1	CCT(342-378aa)	45.33	3.00E-70	42.33	4.00E-63
Medtr_COL	MTR_4g0071811[b]	393	*Medicago truncatula*	RHN64690.1	CCT(342-384aa)	45.78	2.00E-75	42.64	3.00E-68
Nicat_CIL-I	LOC109238457 A4A49_06707[c]	428	*Nicotiana attenuata*	XP_019260475.1	CCT(373-415aa)	43.82	2.00E-85	42.59	7.00E-82
Nicat_CIL-II	LOC109238456 A4A49_06709[c]	385	*Nicotiana attenuata*	XP_019260474.1	-	39.91	1.00E-66	41.36	2.00E-67
Nicsy_CIL-I	LOC104229215	399	*Nicotiana sylvestris*	XP_009780110.1	-	42.28	5.00E-67	40.83	3.00E-68
Nicsy_CIL-II	LOC104246955	424	*Nicotiana sylvestris*	XP_009801175.1	CCT(373-416aa)	43.31	3.00E-83	42.35	8.00E-81
Nicta_CIL	LOC107760235	425	*Nicotiana tabacum*	XP_016433772.1	CCT(370-412aa)	44.62	3.00E-88	43.5	2.00E-86

Nicto_CIL	LOC104090569	425	*Nicotiana tomentosiformis*	XP_009593986.1	CCT(370-413aa)	44.84	6.00E-89	43.74	4.00E-87
Orybr_CIA2-I	LOC102710464<br>OB02G13170[c]	480	*Oryza brachyantha*	XP_006646882.1	CCT(426-469aa)	37.55	1.00E-34	39.74	9.00E-37
Orybr_CIA2-II	LOC102703227<br>OB06G33920[c]	432	*Oryza brachyantha*	XP_015693696.1	CCT(371-415aa)	29.02	2.00E-28	31.28	2.00E-32
Orygla_CCT protein-I	ORGLA02G0034600[c]	484	*Oryza glaberrima*	UniProtKB-I1NX90	CCT(430-472aa)	61.61	7.00E-34	33.19	8.00E-47
Orygla_CCT protein-II	ORGLA06G0219500[c]	466	*Oryza glaberrima*	UniProtKB-I1Q4Y2	CCT(412-454aa)	29.3	7.00E-31	29.64	6.00E-28
OsI_CMF3[a]	OsI_05849<br>BGIOSGA007536[c]	474	*Oryza sativa (Indica Group)*	EAY84475.1	CCT(430-463aa)	54.46	3.00E-25	31.5	1.00E-37
OsI_CMF7[a]	OsI_24346<br>BGIOSGA023549[c]	466	*Oryza sativa (Indica Group)*	EEC81257.1	CCT(416-454aa)	29.3	7.00E-31	29.64	6.00E-28
OsJ_CMF3[a]	LOC4328296<br>Os02g0148000[c]	482	*Oryza sativa (Japonica Group)*	XP_015624719.1	CCT(428-471aa)	61.61	7.00E-34	33.41	2.00E-46
OsJ_CMF7[a]	LOC4341958<br>Os06g0699600[c]	466	*Oryza sativa (Japonica Group)*	XP_015644406.1	CCT(416-454aa)	29.07	8.00E-30	29.4	6.00E-27
Phavu_CCT protein	PHAVU_003G141200g	380	*Phaseolus vulgaris*	XP_007154712.1	CCT(340-378aa)	42.47	2.00E-72	43.48	7.00E-69
Phavu_hypothetical protein	PHAVU_009G236100g	264	*Phaseolus vulgaris*	XP_007138773.1	-	29.19	4.00E-23	27.93	4.00E-16
Setit_CIA2	LOC101763382<br>SETIT_006393mg[c]	459	*Setaria italica*	XP_004966116.1	-	25.6	4.00E-15	45.21	1.00E-13
Setit_CMF3[a]	LOC101777609<br>SETIT_017125mg[c]	483	*Setaria italica*	XP_012698033.2	CCT(429-472aa)	34.75	2.00E-54	33.61	2.00E-51
Solch_CCT protein	EJD97_009489	432	*Solanum chilense*	TMW95010.1	CCT(381-423aa)	44.16	8.00E-91	43.35	8.00E-85
Solly_CIA2	LOC101255506<br>Solyc07g008540.3[c]	432	*Solanum lycopersicum*	XP_004242804.1	CCT(381-424aa)	44.35	5.00E-91	43.12	2.00E-84
Solpe_CIA2	LOC107026098	431	*Solanum pennellii*	XP_015082438.1	CCT(380-423aa)	44.01	5.00E-92	43.22	6.00E-87
Soltu_CIL	LOC102605364[b]<br>PGSC0003DMG400022983[c]	432	*Solanum tuberosum*	XP_006362450.1	CCT(381-424aa)	45.72	9.00E-92	42.2	6.00E-82
Sorbi_CMF3[a]	LOC8074627<br>SORBI_3004G040100[c]	486	*Sorghum bicolor*	XP_002451540.1	CCT(432-475aa)	33.8	4.00E-52	44.29	4.00E-11
Sorbi_CIA2	LOC8065562<br>SORBI_3010G250100[c]	454	*Sorghum bicolor*	XP_021304472.1	-	40.3	3.00E-09	34.38	2.00E-51
Triae_CCT protein-I	TraesCS6A02G118000[c]	492	*Triticum aestivum*	UniProtKB-A0A3B6NM01	CCT(438-480aa)	32.48	2.00E-46	32.45	7.00E-48
Triae_CCT protein-II	TraesCS6B02G146200[c]	484	*Triticum aestivum*	UniProtKB-A0A3B6PIL6	CCT(440-482aa)	30.57	8.00E-38	31.3	3.00E-39
Triae_CCT protein-III	TraesCS6D02G108000[c]	494	*Triticum aestivum*	UniProtKB-A0A3B6QBQ3	CCT(440-482aa)	32.15	2.00E-47	31.39	9.00E-49
Triae_CCT protein-IV	TraesCS7A02G422200[c]	458	*Triticum aestivum*	UniProtKB-A0A3B6RKZ5	CCT(404-446aa)	29.12	5.00E-25	28.68	7.00E-28
Triae_CCT protein-V	TraesCS7B02G322600[c]	457	*Triticum aestivum*	UniProtKB-A0A3B6SPQ1	CCT(403-445aa)	27.69	1.00E-23	27.76	5.00E-26

Triae_CCT protein-VI	TraesCS7D02G414400[c]	457	*Triticum aestivum*	UniProtKB - A0A3B6TPS5	CCT(403-445aa)	27.4	5.00E-23	28.64	2.00E-25
Tritu_CCT protein-I	TRITD_6Av1G037420[bc]	482	*Triticum turgidum subsp. duru*	VAI43762.1	CCT(438-480aa)	31.67	6.00E-42	31.62	2.00E-43
Tritu_CCT protein-II	TRITD_6Bv1G050550[bc]	501	*Triticum turgidum subsp. duru*	VAI55361.1	CCT(440-482aa)	31.46	9.00E-42	32.23	3.00E-43
Tritu_CCT protein-III	TRITD_7Av1G227500[bc]	453	*Triticum turgidum subsp. duru*	VAI79113.1	CCT(399-441aa)	29.12	5.00E-25	28.68	6.00E-28
Tritu_CCT protein-IV	TRITD_7Bv1G176630[bc]	457	*Triticum turgidum subsp. duru*	VAI91171.1	CCT(403-445aa)	27.69	1.00E-23	27.76	5.00E-26
Zeama_Uncharacterized protein-I	LOC100501277 Zm00001d014664[c]	435	*Zea mays*	NP_001353852.1	-	26.7	4.00E-14	26.91	5.00E-18
Zeama_CIA2-I	LOC103626617 Zm00001d015268[c]	485	*Zea mays*	XP_008645243.1	CCT(431-474aa)	33.74	5.00E-50	33.96	1.00E-50
Zeama_Uncharacterized protein-II	LOC100384273 Zm00001d036485[c]	443	*Zea mays*	NP_001170309.2	-	26.24	2.00E-17	26.37	5.00E-17
Zeama_CIA2-II	LOC100281423 Zm00001d053880[c]	459	*Zea mays*	XP_008677196.1	CCT(405-448aa)	63.72	6.00E-35	25.11	2.00E-12

[a] CMF proteins, as per Cockram et al. (2012).
[b,c] Gene symbol and locus refer to the NCBI or Gramene databases.
[d] CCT-motif fragment, as predicted by PROSITE (prosite.expasy.org).
[e] Identity value and E-value from the NCBI database.
