# Supplementary material for: Sequence analysis and protein interactions of Arabidopsis CIA2 and CIL proteins
Source: Bot Stud. 2020 Jun 18;61:20. doi: 10.1186/s40529-020-00297-z (PMC7303255; doi:10.1186/s40529-020-00297-z)
Supplement: Supplementary file 2 — Additional file 2: Figure S1. CC1 ~ 5 motifs conserved between CIA2 and CIL proteins, as predicted by MEME suite. [file 40529_2020_297_MOESM2_ESM.pdf]

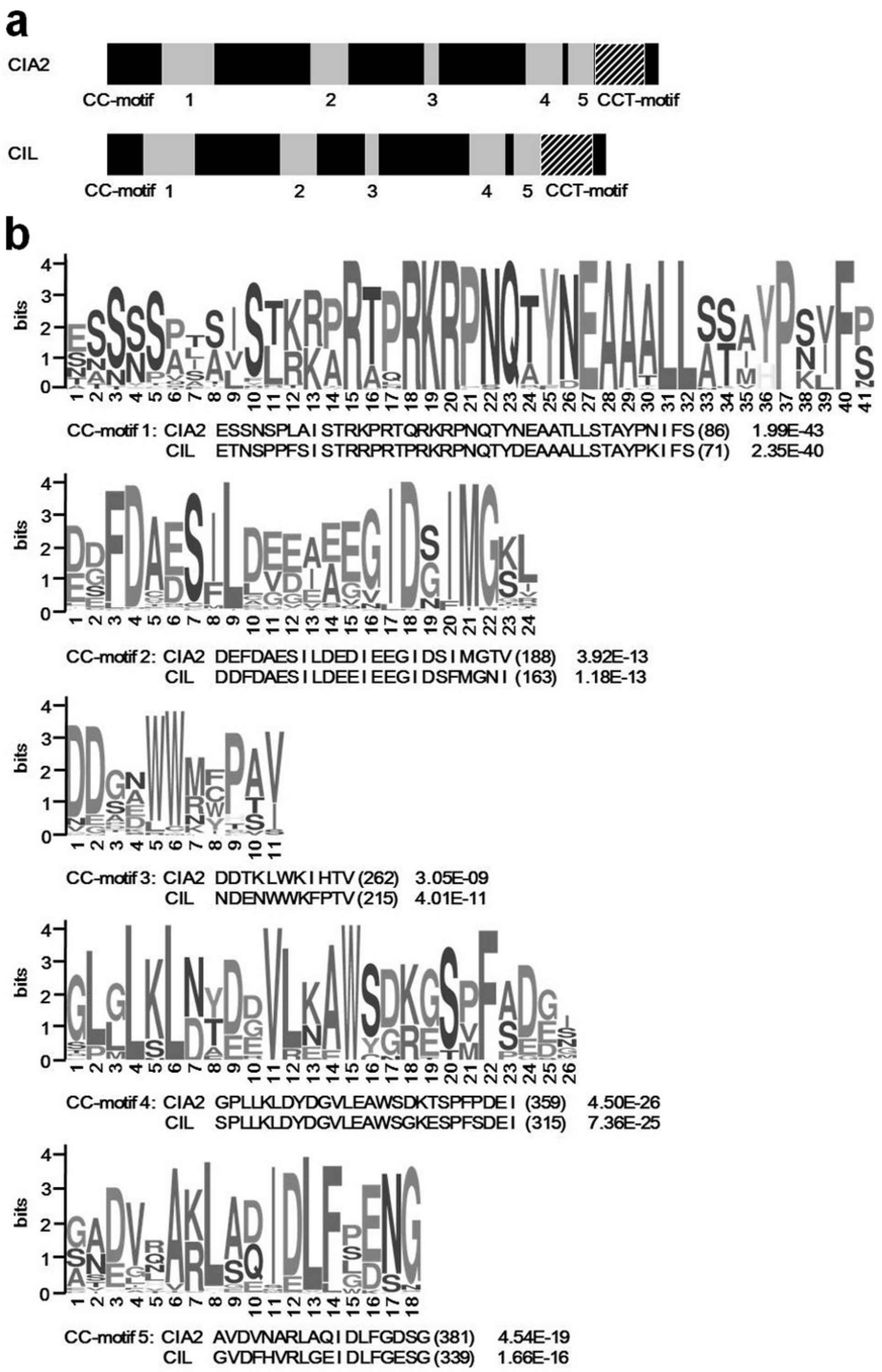

Figure S1. CC1~5 motifs conserved between CIA2 and CIL proteins, as predicted by MEME suite. a) Positions of five predicted motifs in CIA2 and CIL. Grey box, conserved motif. Hatched box, CCT motif. b) Sequence logos of each motif, showing the likelihood of each observed residue at each position. All five motifs are highly significant. The sequences and E-values are shown for each motif.
