# Supplementary material for: Sequence analysis and protein interactions of Arabidopsis CIA2 and CIL proteins
Source: Bot Stud. 2020 Jun 18;61:20. doi: 10.1186/s40529-020-00297-z (PMC7303255; doi:10.1186/s40529-020-00297-z)
Supplement: Supplementary file 3 — Additional file 3: Figure S2. Comparison of CCT motifs of Arabidopsis CIA2, CIL, CO, COL, and TOC1 proteins. [file 40529_2020_297_MOESM3_ESM.pdf]

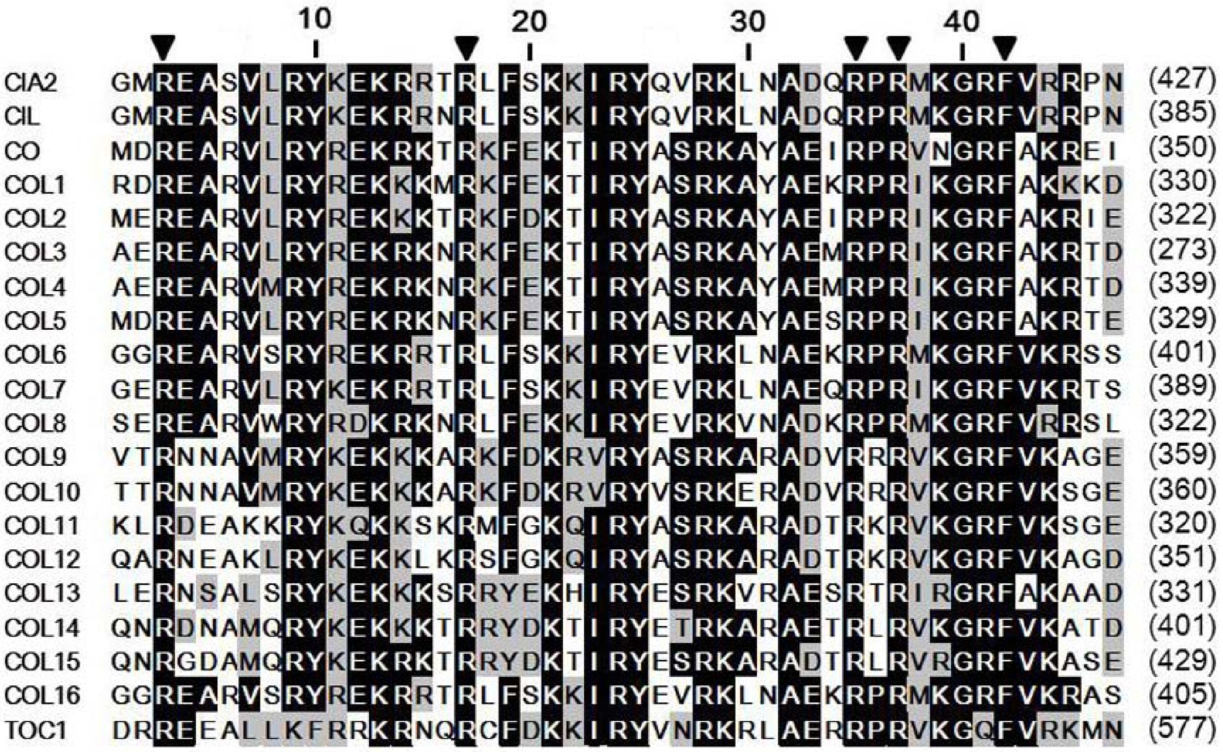

Figure S2. Comparison of CCT motifs of Arabidopsis CIA2, CIL, CO, COL, and TOC1 proteins. The MEGA-X software was used to align these sequences. Numbers in brackets at the end of each sequence reflects the corresponding aa number of each protein sequence. Numbers above the alignment indicate amino acid position (in the alignment). Black and gray backgrounds represent conserved or similar residues, respectively. The arrows indicate conserved residues important for protein-protein interaction.
